# Commentary on the NICE guideline on renal replacement therapy and conservative management

**DOI:** 10.1186/s12882-021-02461-4

**Published:** 2021-08-20

**Authors:** Kunaal Kharbanda, Osasuyi Iyasere, Fergus Caskey, Matko Marlais, Sandip Mitra

**Affiliations:** 1 Brighton and Sussex University Hospitals NHS Trust, Brighton, UK; 2 Manchester Academic Health Science Centre, The University of Manchester, Manchester, UK; 3 John Walls Renal Unit, Leicester General Hospital, Leicester, UK; 4 Bristol Medical School: Population Health Sciences, University of Bristol, Bristol, UK; 5 Richard Bright Renal Unit, Southmead Hospital, Bristol, UK; 6 Great Ormond Street Hospital for Children NHS Foundation Trust, London, UK; 7 UCL Great Ormond Street Institute of Child Health, London, UK; 8 NIHR Devices for Dignity Healthcare Technology Co-Operative, Royal Hallamshire Hospital, Sheffield, UK

## Abstract

NICE Guideline NG107, “Renal replacement therapy and conservative management” (Renal replacement therapy and conservative management (NG107); 2018:1–33) was published in October 2018 and replaced the existing NICE guideline CG125, “Chronic Kidney Disease (Stage 5): peritoneal dialysis” (Chronic kidney disease (stage 5): peritoneal dialysis | Guidance | NICE; 2011) and NICE Technology Appraisal TA48, “Guidance on home compared with hospital haemodialysis for patients with end-stage renal failure”(Guidance on home compared with hospital haemodialysis for patients with end-stage renal failure (Technology appraisal guideline TA48); 2002) The aim of the NICE guideline (NG107) was to provide guidance on renal replacement therapy (RRT), including dialysis, transplant and conservative care, for adults and children with CKD Stages 4 and 5. The guideline is extremely welcomed by the Renal Association and it offers huge value to patients, clinicians, commissioners and key stakeholders. It overlaps and enhances current guidance published by the Renal Association including “Haemodialysis” (Clinical practice guideline: Haemodialysis; 2019) which was updated in 2019 after the publication of the NICE guideline, “Peritoneal Dialysis in Adults and Children” (Clinical practice guideline: peritoneal Dialysis in adults and children; 2017) and “Planning, Initiation & withdrawal of Renal Replacement Therapy” (Clinical practice guideline: planning, initiation and withdrawal of renal replacement therapy; 2014) (at present there are no plans to update this guideline).

There are several strengths to NICE guideline NG107 and we agree with and support the vast majority of recommendation statements in the guideline. This summary from the Renal Association discusses some of the key highlights, controversies, gaps in knowledge and challenges in implementation. Where there is disagreement with a NICE guideline statement, we have highlighted this and a new suggested statement has been written.

## Main text

### Summary of recommendations

#### Indications for starting dialysis

**Table:** 

1.1 Indications for starting dialysis	
1.1.1 Follow the recommendations on referral criteria in NICE’s guideline on chronic kidney disease in adults 1.1.2 Consider starting dialysis when indicated by the impact of symptoms of uraemia on daily living, or biochemical measures or uncontrollable fluid overload, or at an estimated glomerular filtration rate (eGFR of around 5 to 7 ml/min/1.732 if there are no symptoms. 1.1.3 Ensure the decision to start dialysis is made jointly by the person (or, where appropriate, their family members or carers) and their healthcare team. 1.1.4 Before starting dialysis in response to symptoms, be aware that symptoms may be caused by non-renal conditions	

We fully support and endorse this section of the guidance. Symptoms and eGFR should be taken into account but with some caution if waiting to start until someone is very symptomatic as this could impact on patient wellbeing, education and training for self-care or shared care. Systems and tools such as patient-reported outcome measures (PROMs) could be used to collect and monitor the severity of symptoms reported by patients for an optimal start of RRT.

In infants and children there are no data to support starting dialysis on the basis of eGFR alone [1]. Decisions to start dialysis should be on the basis of symptoms which include those listed in the NICE guidance but also include poor growth and nutrition which are critical in this early stage of life [2]. Using eGFR to decide when to initiate dialysis is particularly challenging in infants and children under 2 years of age, where rapid growth and ongoing renal maturation make it difficult to estimate GFR.

#### Preparing for renal replacement therapy or conservative management

**Table:** 

**1.2. Preparing for renal replacement therapy or conservative management**	
1.2.1 Start assessment for renal replacement therapy (RRT) or conservative management 1 year before therapy is likely to be needed, including for those with a failing transplant 1.2.2 Involve the person and their family members or carers (as appropriate) in shared decision-making over the course of assessment to include: • Clinical preparation • Psychosocial evaluation, preparation and support • The individuals preferences for type of RRT and when to start • How decisions are likely to affect daily life 1.2.3 Consider further assessment by a clinical psychologist or psychiatrist for: • All children and young people being considered for a transplant, and • Adults being considered for a transplant if risk factors for poor outcomes have been identified; these may include: o lack of social support o neurocognitive illness o non-adherence (medicines, diet, hospital appointments) o poor understanding of process and complexities of treatment o poorly controlled mental health conditions or severe mental illness o substance misuse or dependence	

We feel strongly that decisions regarding RRT modality or conservative care should be fully individualised and should take into consideration all of the factors mentioned in this section of the NICE guideline. All treatment options (dialysis, transplant and conservative care) should be discussed with patients (and families or carers for those under 18), including home dialysis. Patient autonomy, involvement and choice have been associated with favourable outcomes on RRT [3]. It remains unclear how clinical factors, demographics (age) and patient functional status impact on the choice and outcomes of RRT and conservative care.

#### Choosing modalities of renal replacement therapy or conservative management

**Table:** 

1.3 (A) Choosing modalities of renal replacement therapy or conservative management	
*1.3.1 Offer a choice of RRT or conservative management to people who are likely to need RRT* *1.3.2 Ensure that decisions about RRT modalities or conservative management are made jointly with the person (or with their family members or carers for children or adults lacking capacity) and healthcare team, taking into account:* • *Predicted quality of life* • *Predicted life expectancy* • *The person’s preferences (see recommendations in section 1.8)* • *Other factors such as co-existing conditions* *1.3.3 Offer people (and their family) members or carers, as appropriate) regular opportunities:* • *To review the decision regarding RRT modalities or conservative management* • *To discuss any concerns or changes in their preferences*

**Table:** 

1.3 (B) Transplantation	
*Transplantation* *1.3.4 Discuss the individual factors that affect the risks and benefits of transplantation with all people who are likely to need RRT, and their family members or carers (as appropriate)* *1.3.5 Include living donor transplantation in the full informed discussion of options for RRT* *1.3.6 Offer pre-emptive living donor transplant (where there is a suitable living donor) or pre-emptive listing for deceased donor transplantation to people considered eligible after a full assessment* *1.3.7 Do not exclude people from receiving a transplant based on BMI alone*

**Table:** 

1.3 (C) Choice of dialysis modalities	
*1.3.8 Offer a choice of dialysis modalities at home or in centre ensuring that the decision is informed by clinical considerations and patient preferences (see recommendation 3.2)* *1.3.9 (NICE) Offer all people who choose peritoneal dialysis a choice of continuous ambulatory peritoneal dialysis (CAPD) or automated peritoneal dialysis (APD), if this is medically appropriate.* **1.3.9 (RA) We recommend that adults who have opted for PD be offered APD or CAPD according to their preference, if clinically feasible. We suggest that assisted PD be made available as a viable option, for those who cannot undergo self-care PD.** *1.3.10 Consider peritoneal dialysis as the first choice for children 2 years or under* *1.3.11 (NICE) Consider HDF rather than HD in centre (hospital or satellite)* *Consider HDF or HD at home, taking into account the suitability of the space and facilities* ***1.3.11 (RA) We recommend that either high flux HD or HDF can be offered as an RRT modality both in-centre or at home, taking into account the local infrastructure and technology available.***	

**i) Peritoneal Dialysis**

The flexibility offered by APD during daytime hours has led to an expansion in its use over time [4], with 59% of the UK PD population on APD [5]. The current clinical evidence comparing outcomes between continuous ambulatory peritoneal dialysis (CAPD) and automated peritoneal dialysis (APD) is of low grade and is largely based on observational studies that are limited by confounding and bias and may not always be relevant to the NHS population. The randomised studies [6, 7] were invariably underpowered to detect significant differences between CAPD and APD. Consequently, one modality was not found to be consistently superior to the other in terms of patient survival, technique survival or health-related quality of life. These small RCTs from the 1990s reported lower peritonitis rates with APD as compared to CAPD. In the more recent, albeit observational studies, the reported outcomes are inconsistent. The NICE guidance is consistent with the ISPD update on peritonitis in 2016 in suggesting that the risk of peritonitis should not determine PD modality choice [8]. Clinical outcomes therefore no longer drive the choice of modality (CAPD vs APD) in adults opting for peritoneal dialysis, with patient preference being the principal determining factor.

The studies included in the NICE guidance evidence review did not include patients on staff assisted PD (aPD). However, aPD is increasingly used to facilitate dialysis at home in patients, often older and frail, who are not capable of self-care PD. Observational studies have found that aPD is associated with comparable clinical outcomes when compared in-centre haemodialysis in older people [9, 10]. In comparison to self-care PD, aPD has been shown to have similar quality of life outcomes [11] and a lower risk of technique failure [12]. A recent retrospective study of 6167 patients from the French PD registry found that there is no difference in technique survival and peritonitis risk between assisted APD and assisted CAPD [13].

There are cost implications associated with the utilization of aPD, with very limited evidence on its cost effectiveness. Future research should include evaluation of the health economic impact of aPD in comparison to other renal replacement modalities. A cost effectiveness analysis of the various aPD delivery models (assisted APD and assisted CAPD) would add value to the current body of evidence.

We agree with the NICE guidance that clinicians should consider PD as the first choice for children 2 years or under. PD is the commonest dialysis modality in children, accounting for 45% of patients starting RRT in the UK in 2016 [14]. Clinical, patient and family factors, as well as age predominantly determine the choice of dialysis modality in the paediatric population, with PD being predominant in those aged 5 years or below [15]. The perceived benefits of PD in this age group are the flexibility of dialysing at home as well as preserving vascular access. There is, however, a lack of evidence on comparative outcomes between HD and PD in this cohort.

**ii) Home Haemodialysis**

Home haemodialysis (HHD) remains an under-utilised modality in the UK [16] despite it being a therapy associated with lower costs compared with in-centre haemodialysis [17–23]. HHD allows greater flexibility in treatment, a considerable reduction in travel to hospital and enables more extended and frequent prescriptions which are associated with several clinical benefits including a reduction in LV mass [24, 25], improved blood pressure control [24, 26, 27], improved phosphate clearance [28, 29] and lower ultrafiltration rates. Several observational studies have demonstrated a significant survival advantage associated with HHD [30–32] although prospective randomised studies supporting this finding are lacking.

There have been no randomised trials comparing home to hospital dialysis outcomes. Patients choosing HHD however, go through steps of clinical selection, education and rigorous training on initiation of RRT. The impact of such interventions, hitherto untested in dialysis clinical trials, might provide insight into reported improvements in patient outcomes in HHD when compared to in-centre HD [32]. It is clear that patients frequently do not always start on their chosen RRT modality [33, 34] and that this can be a key barrier to the uptake of home therapies. Effective tool and strategies should therefore be put in place to minimise these barriers in order to increase the proportion of patients starting on home modalities.

Like PD, the choice of HHD is largely determined by patient choice and training. The home setting, flexible scheduling, dialysis intensity, lower pill burden and freedom with diet and fluids offer major advantages to those who choose HHD. Although many of the benefits observed with HHD may be attributed to patient selection and preparation, given the numerous benefits reported in the literature, we feel that this treatment modality should be considered and offered to all patients deemed suitable.

Several centres in the UK report the use of HDF in the home setting [33] where there is local provision and technical feasibility for offering this therapy. Offering the same HD modality in-centre and in the home setting allows for continuity of care and facilitates smoother transition from hospital to home dialysis. There is very little published literature on the safety of HDF in the home setting [34]. There are no data to suggest that HDF is unsafe in the home setting provided HDF devices are installed, maintained and used as instructed and that feed water is monitored at least every 6 months for chemical and microbial quality [35]. Whilst there is no clear reason for the benefits of either HD or HDF to be any different at home compared with in-centre, the effects of more frequent and extended prescriptions using high volume HDF are largely unknown and under researched. We agree that at present there is insufficient evidence to recommend one modality over the other in the home setting and that either HD or HDF can be considered.

**iii) In-Centre Haemodialysis**

High flux haemodialysis is predominantly a diffusive treatment combined with limited volumes of convective clearances. Haemodiafiltration (HDF), combines both diffusive and high dose convective therapy. Newer technology has enabled ultrapure replacement solution to be generated and delivered by the device (on-line HDF), allowing higher convective volumes and easier delivery of this therapy to patients. As a result, there has been a growth in the use of HDF as a treatment modality [36], particularly in Europe. However, there is considerable geographic variation in uptake [37]. There is also increasing use of HDF in children and adolescents [38].

There have been several recent prospective randomised clinical trials comparing HDF with HD treatment. Of the 4 large recent studies (CONTRAST study [39], ESHOL study [40], the FRENCHIE study [41] and the Turkish study [42]), only a single trial (ESHOL) has demonstrated a benefit of HDF over HD in terms of the primary outcome measure. Evidence from this trial needs to be interpreted with caution, however, as discussed below.

NICE’s conclusion that HDF was associated with an 18% reduction in mortality (relative risk (RR) 0.82, 95% confidence interval (95% CI) 0.72–0.94) was unexpected, as at least three systematic reviews had recently reported that there was no evidence of superiority of HDF over HD [39–41]. An investigation reproducing their analyses found that the explanation was two-fold.

First, NICE had used a fixed effects model. Such an approach assumes that the effect of an intervention is in the same direction and of similar magnitude in all the studies being included in the meta-analysis. This was not the case for trials of HDF vs HD and a more appropriate approach to minimise type 1 error and inappropriately narrow confidence intervals would have been a random effects model [43, 44]. When this was applied, the effect of HDF became non-significant (RR 0.84, 95% CI 0.64–1.10). Second, they took no account of biases within some of the trials that were driving the effect. Specifically, two trials [40, 42] removed about 10 % of patients after randomisation from the HDF arm due to the inability to achieve high volumes of HDF in these patients (patients with similar blood flow issues in the HD arm were not removed). The key determinants of high convective volumes (filtration fraction, blood flow and treatment time) favour patients with more optimal vascular access and less comorbidity in whom outcomes may already be superior. This is reflected by the imbalance of age, diabetes and catheter use in the ESHOL study [40]. Combining biased studies in a meta-analysis amplifies the bias, with no way to weight biased studies differently and reduce their influence on the observed effect. Instead, therefore, it is recommended that sensitivity analyses are done excluding the biased trials, to see how much they are driving the effect. Excluding the two trials that reported excluding patients post randomisation from the HDF arm [40, 42] from the NICE meta-analysis resulted in complete loss of any evidence of a benefit of HDF over HD (RR 0.94, 95% CI 0.53–1.66).

Recognising this, feedback from the Renal Association, British Renal Society and Cochrane Renal challenged the draft NICE recommendation and NICE changed its recommendation to “consider HDF”. The authors feel the current NICE recommendation to consider HDF over high flux HD in-centre is not supported by credible evidence and that further evidence is needed. There are currently two large randomised controlled trials underway (H4RT (https://www.cochranelibrary.com/central/doi/10.1002/central/CN-01891367/full) and the CONVINCE study (https://www.trialregister.nl/trial/6942)) which have been designed to compare HD with HDF with a target convective volume of 21+ litres. Additionally, the MoTHER HDx study (https://clinicaltrials.gov/ct2/show/NCT03714386), comparing medium cut-off haemodialysis with HDF is also underway. Awaiting the results of these large and significant studies (target recruitment 3350 participants combined for HDF vs HD) will allow a much more informed recommendation.

The field of dialysis is rapidly advancing with trials in new technology, medium cut-off membranes, miniaturised devices and alternative modalities (incremental, alternate day and nocturnal dialysis) Further technical guidance is available in the Renal Association guideline, “Haemodialysis” [45] including comprehensive evidence-based practice guideline on haemodialysis prescribing including scheduling time and frequency to improve patient outcomes.

**iv) Transplantion**

**Table:** 

1.3 (B) Transplantation	
*Transplantation* *1.3.12 Discuss the individual factors that affect the risks and benefits of transplantation with all people who are likely to need RRT, and their family members or carers (as appropriate)* *1.3.13 Include living donor transplantation in the full informed discussion of options for RRT* *1.3.14 Offer pre-emptive living donor transplant (where there is a suitable living donor) or pre-emptive listing for deceased donor transplantation to people considered eligible after a full assessment* *1.3.15 Do not exclude people from receiving a transplant based on BMI alone*	

We fully support and endorse this section of the guidance. We agree that robust evidence is needed in determining the optimal timing for renal transplantation. Pre-emptive renal transplantation (a mode of transplantation that lends itself to pre-planning) is considered to be the preferred initial option for RRT in eligible patients. We suggest, however, that the existing evidence does not support earlier pre-emptive kidney transplant on the basis of GFR. A matched cohort study from the Australian and New Zealand Dialysis and Transplant (ANZDATA) registry, did not find a statistically significant difference in survival between pre-emptive (median GFR of 9.6 ml/min/1.73 m2) and non-pre-emptive live kidney transplant recipients with up to 6 months HD vintage (median GFR of 6.9 ml/min/1.73 m2), even when lead time bias was considered [46]. An earlier cohort study of 19,471 pre-emptive transplant recipients reported to United Network of Organ Sharing (UNOS) found no association between the GFR at the time of transplantation and patient or graft survival [47].

In the absence of evidence to the contrary, listing potential recipients for transplantation 6 months prior to anticipated start of RRT appears to be a sensible approach.

**v) Conservative management**

There is considerable variability in the uptake of conservative management both within the UK [48] and globally [49, 50] and national registry data is lacking for this modality is most countries. When considering the options of conservative management and dialysis, decision-making can be difficult and there are no randomised studies comparing outcomes between patients choosing conservative management and dialysis. Data from observational studies [51] suggest comparable survival in older patients [52] and those with significant co-morbidities [53] or poor performance status. Given the nature of these studies, there is risk of significant bias as well as confounding factors which makes their interpretation difficult and may in part explain the variability seen in current clinical practice. In addition to survival, the influence of treatment modality choice on other factors such as measures of quality of life, the number of hospital-free days, symptom burden, travel and the effect on family and carers should be considered and discussed. Once again, high quality data in this area is lacking and should be a focus for future research. There is currently one large randomised controlled trial examining this topic in the UK (The Prepare for Kidney Care Study https://www.cochranelibrary.com/central/doi/10.1002/central/CN-01887674/full).

We fully support the NICE NG107 guideline in offering conservative management as a treatment option alongside RRT modalities and that decision-making should be made in conjunction with the patient and carers or family members where appropriate. Further technical guidance within this field is available in the Renal Association guideline, “Planning, Initiating and Withdrawal of Renal Replacement Therapy” [54].

#### Planning dialysis access formation

**Table:** 

1.4 Planning dialysis access formation	
1.4.1 Discuss with the person, their family members and carers (as appropriate) the risk and benefits of the different types of dialysis access, for example, fistula, graft, central venous or peritoneal dialysis catheter 1.4.2 (NICE) When peritoneal dialysis is planned via a catheter placed by an open surgical technique, aim to create the access around 2 weeks before the anticipated start of dialysis. **1.4.2 (RA) We recommend a break in period of at least 2 weeks after PD catheter insertion, taking into consideration patient preference and local clinical pathways to avoid the need for temporary HD. We suggest that low volume APD be used in the setting of acute start PD.** 1.4.3 When HDF or HD is planned via an arteriovenous fistula, aim to create the fistula around 6 months before the anticipated start of dialysis to allow for maturation. When deciding to timing, take into account the possibility of the first fistula failing or needing further interventions before use 1.4.4 Offer ultrasound scanning to determine vascular access sites for creating arteriovenous fistulae for HDF or HD	

The NICE recommendations on access planning are broadly supported by the authors. They highlight optimal timing of access placement to avoid unplanned RRT initiation by temporary vascular access, which is associated with adverse clinical outcomes.

Whilst observational studies suggest better outcomes in terms of access patency and mortality with early as against late arteriovenous fistula formation in potential HD patients, the optimal time for access placement differs depending on the outcome measure evaluated. Pragmatically, it is difficult to predict with certainty the timeframe for HD initiation due to unpredictable clinical events and non-linear GFR decline. It is therefore unsurprising that the recommendations on timing of AVF placement differ among the various national bodies [55], with some opting for GFR based criteria as against time. In the absence of robust evidence, the NICE recommendation for access formation at about 6 months prior to intended use, seems reasonable taking local clinical pathways into consideration.

Peritoneal access placement is a key part of the pathway for preparing a person for PD. There is significant variation in catheter insertion methods across renal units, based on local facilities and expertise. A recent systematic review involving 7 cohort studies found that the advanced laparoscopic insertion technique was associated with clinical superior outcomes when compared to open surgical insertion, including catheter migration, survival and leaks [56]. The NICE recommendation on timing of catheter insertion is predominantly based on a single randomised study of 122 participants which found a higher rate of leaks in those starting PD one week post insertion compared to two and four weeks post insertion. All catheters were inserted using the open surgical technique and thus the findings may not be applicable to other insertion methods [57]. The study may also be statistically underpowered as it was stopped early. The recommendation is thus based on moderate grade evidence. Nevertheless, the recently published ISPD guidance on optimal PD access recommends a break-in period of at least 2 weeks, regardless of the catheter insertion method [58]. This recognises the need to factor in patient convenience, training duration and availability into care pathway for establishing PD access.

On the other hand, there is a role for acute start PD in unplanned starters who would like home dialysis in the long term, avoiding the need for temporary HD. Several observational studies have reported a higher risk of mechanical complications (malfunction, leaks etc.) with urgent start PD (generally less than 2 weeks post insertion) compared to planned start PD. These complications are generally conservatively managed with no impact on patient or technique survival [59, 60]. Low grade observational evidence suggests that clinical outcomes are at least similar when acute start PD is compared to acute start HD [61, 62]. An important modifier of outcomes relating to acute start PD is the use of low volume APD to reduce the risk of leaks. This is a grade 1C recommendation by the ISPD [58].

The optimal break-in period post insertion may vary depending on the method of insertion used. Studies that compare the various insertion methods particularly in acute start PD would be beneficial.

#### Indications for switching or stopping renal replacement therapy

**Table:** 

1.5 Indications for switching or stopping renal replacement therapy	
1.5.1 Offer information on all medically appropriate treatment options when discussing switching RRT modality. 1.5.2 Consider switching treatment modality or stopping RRT if medically indicated or if the person (or, where appropriate, their family members or carers) asks. 1.5.3 Plan switching treatment modality or stopping RRT in advance wherever possible. 1.5.4 Do not routinely switch people on peritoneal dialysis to a different treatment modality in anticipation of potential future complications such as encapsulating peritoneal sclerosis. However, monitor risk factors, such as loss of ultrafiltration. 1.5.5 Seek specialist advice on the need for switching treatment modality when women become pregnant or wish to become pregnant.	

We are in support of the guidance on switching treatment modalities or stopping renal replacement therapy. The recommendation not to electively swap patients on PD to other modalities in anticipation of encapsulating peritoneal sclerosis (EPS) is very much consistent with consensus view as per the ISPD position paper. Whilst longer PD vintage is associated with a higher risk of EPS, evidence of that elective transition from PD is preventative is lacking.

RRT patients are likely to utilise different modalities at different time points of their disease. It is therefore important to consider treatment pathways rather than individual RRT techniques Perspectives of patients, caregivers, and health professionals on the process of transitioning are even less well documented. Available literature suggests that at present, transition between the different modalities is poorly coordinated, causing significant morbidity and mortality [63]. While predictors of PD technique failure and transition to HD have been assessed in some studies, clinical outcomes following transfer from PD to in-centre HD are lacking. HD-to-PD transition, has been associated with an increased risk of death and technique failure [64]. Given that more than one-third of patients will experience a transition to another RRT modality, particularly to facility-based conventional HD within the first 3 years on PD [64], a better understanding of morbidity and mortality associated with this transition is critically important for the care of patients with ESKD.

A key transition point is during hospitalisation and readmissions for both RRT and conservative care patients. Systems for improving communication between the hospital and nephrologist about patient care are needed [65]. Transition considerations as outlined in the guideline are key to address such high-risk periods in patient lives on RRT. Robust policies informed by ongoing research will be required for implementation [64].

#### Recognising symptoms, diet and fluids and information, education and support

**Table:** 

1.6 Recognising Symptoms	
1.6.1 Recognise that people on RRT or receiving conservative management may have the symptoms in Table 1 [1] and that these may affect their day-to-day life. 1.6.2 Throughout the course of RRT and conservative management: • Ask people about any symptoms they have. • Explore whether symptoms are due to the renal condition, treatment or another cause. • Explain the likely cause of the symptoms and how well treatment may be expected to control them.

**Table:** 

1.7 Diet and fluids	
1.7.1 Offer a full dietary assessment by a specialist renal dietitian to people starting dialysis or conservative management. This should include: • weight history • fluid intake • sodium • potassium • phosphate • protein • calories • micronutrients (vitamin and minerals) 1.7.2 After transplantation, offer dietary advice from a healthcare professional with training and skills in this area. 1.7.3 Re-assess dietary management and fluid allowance when: a person’s circumstances change (for example, when switching RRT modality), or biochemical measures or body composition measures (for example, unintentional weight loss) indicate, or the person (or, where appropriate, their family members or carers) asks. 1.7.4 Provide individualised information, advice and ongoing support on dietary management and fluid allowance to the person and their family members or carers (as appropriate). The information should be in an accessible format and be sensitive to the person’s cultural needs and beliefs. 1.7.5 Follow the recommendations on dietary management and phosphate binders in NICE’s guideline on chronic kidney disease (stage 4 or 5): management of hyperphosphataemia.	
1.8 Information, education and support	
1.8.1 To enable people, and their families and carers (as appropriate), to make informed decisions, offer balanced and accurate information about: all treatments available to them (including RRT modalities and conservative management), and how the treatments may affect their lives. 1.8.2 Recognise the psychological impact of a person being offered RRT or conservative management and discuss what psychological support may be available to help with decision-making. 1.8.3 Discuss with people which treatment options are available to them and explain why any options may be inappropriate or not advised. 1.8.4 Offer oral and written information and support early enough to allow time for people to fully understand their treatment options and make informed decisions. Information should be in an accessible format. 1.8.5 Direct people to other sources of information and support (for example, online resources, pre-dialysis classes and peer support). 1.8.6 Remember that some decisions must be made months before RRT is needed (for example, a fistula is created at least 6 months before starting dialysis). 1.8.7 Be prepared to discuss the information provided both before and after decisionsar e made, in line with the person’s wishes. 1.8.8 Take into account information the person has obtained from other sources (suchas family members and carers) and how this information has influenced their decision. 1.8.9 Ensure that healthcare professionals offering information have specialist knowledge about late stage chronic kidney disease and the skills to support shared decision-making (for example, presenting information in a form suitable for developmental stage). 1.8.10 Offer people who have presented late, or who started dialysis in an unplanned way, the same information as people who present at an earlier stage. 1.8.11 Follow the recommendations on enabling patients to actively participate in their care in NICE’s guideline on patient experience in adult NHS services and on information and education in NICE’s guideline on chronic kidney disease in adults.	

We fully support the guidance on recognising symptoms during the course of RRT, providing adequate nutritional support and developing resources and systems to provide adequate information, education and support to patients, carers and family members. Growth failure can be an important manifestation of CKD in the younger population; clinicians should be aware of this and monitor it not only through weight history but also through charting of height and weight on age-appropriate growth charts.

#### Coordinating care

**Table:** 

1.9 Coordinating care	
1.9.1 Provide the person with the contact details of the healthcare professional responsible for their overall renal care: • before they start RRT or conservative management • when they switch from one modality to another. 1.9.2 Coordinate care to reduce its effect on day-to-day life and wellbeing (treatment burden). For example, take account of people’s preferences and avoid scheduling appointments on non-dialysis days for people on hospital dialysis wherever possible. 1.9.3 Follow the recommendations on: • delivering an approach to care that takes account of multimorbidity in NICE’s guideline on multimorbidity, and • continuity of care and relationships, and enabling patients to actively participate in their care in NICE’s guideline on patient experience in adult NHS services.	

The emphasis on coordination of care in Guidance 1.9 highlights the complex medical needs of this diverse, high-risk patient population. Its implementation is critically dependent on the interface between care pathways and multiprofessional stakeholders (ie. dieticians, specialist nurses providing education, psychologists, diabetes specialists and other key specialists). Care fragmentation in dialysis patients between nephrology units and primary care providers is well recognised and can result in: a) duplication of care leading to overuse, medication errors and scheduling errors, b) uncoordinated care with lack of communication and c) delayed or undelivered care resulting in delays or missed opportunities [66]. Several gaps in care delivery of RRT patients such as vaccination, cancer screening, HbA1c, foot care and eye testing, could be improved through better coordination between primary and secondary care. Considerable growth in the ESKD prevalent population with increasing age, diabetes and multimorbidity and a shrinking nephrology and primary care workforce is predicted [66]. Coordination of primary and secondary care therefore remains a major area of concern. A future dialysis care model will require innovative pathways designed through collaboration of dialysis clinics, nephrologists, GP practices and other secondary care providers to address the needs of this unique patient group. This could deliver major transformation in care through improvements in patient experience, clinical outcomes and efficiency.

Renal replacement therapy for children continues to be co-ordinated through the 13 paediatric nephrology centres in the UK. Shared care arrangements with secondary paediatric services are variable across the regions of the UK, and improved network working is likely to improve patient experience and may improve clinical outcomes. This is particularly important for adolescents approaching transition to adult services, where good co-ordination between paediatric and adult nephrology units is key to ensuring an effective individualised approach [67].

### Audit measures

1. Percentage of patients commencing RRT referred < 3 months and < 12 months before date of starting RRT
2. Percentage of incident RRT patients followed up for > 3 months in dedicated pre- dialysis or low clearance clinic
3. Proportion of incident patients on UK transplant waiting list at RRT initiation
4. Proportion of incident RRT patients transplanted pre-emptively from living donors and deceased donors
5. Proportion of incident patients commencing peritoneal or home haemodialysis
6. Proportion of incident children under 2 years of age commencing peritoneal or haemodialysis
7. Proportion of patients who have undergone a formal education programme prior to initiation of RRT
8. Proportion of incident RRT patients who report that they have been offered a choice of RRT modality
9. Proportion of patients remaining on initial treatment modality 3 and 12 months post initiation of RRT
10. Proportion of patients recording satisfaction with initial RRT decision at 3 and 12 months post initiation of RRT
11. Proportion of patients who have initiated dialysis in an unplanned fashion who have undergone formal education by 3 months.
12. Evidence of formal continuing education programme for patients on dialysis
13. Proportion of planned initiations with established access or pre-emptive transplantation
14. Inpatient/outpatient status of planned initiations
15. eGFR at start of renal replacement therapy
16. The number of patients with Stage 5 CKD who are undergoing conservative kidney management - as a proportion of all patients with Stage 5 CKD
17. The number of patients withdrawing from dialysis as a proportion of all deaths on dialysis
18. Morbidity and mortality associated with transition from Home to Hospital modalities and between modalities in RRT patients
19. Hospitalisation and Readmission rates in RRT
20. Vaccination rates in RRT patients
21. Coordination of care in diabetes in RRT patients
22. Coordination of care - Management of ischemic heart disease in Dialysis

## Conclusion

The NICE guidance on RRT focusses on the entire life course of the patient with ESKD. The focus is on integrated, multidisciplinary and holistic care improvements to meet the needs of this unique, complex and high-risk patient group. Many of the aspects such as care coordination and transition are unique in the guidance. Implementation of these recommendations will require comprehensive review of policies, practice, care pathways and infrastructure, which can be potentially challenging within the constraints of current health care systems. There are several areas of controversy where definitive trial evidence is lacking. Much of our clinical current practice and current guidance is based on expert opinion and data largely obtained from observational studies. High quality prospective randomised studies are needed to answer many questions raised within the areas covered by the NICE guideline. Several of these are already underway, led by the UK kidney community, but further broadening of attitudes towards recognising uncertainty and offering randomisation could transform our ability to generate robust evidence to inform shared decision making. Other NICE research recommendations aimed at improving the gaps in evidence base, will need to be supported by kidney research consortiums and funding bodies. Ongoing trials and recommended research in RRT aim to improve the evidence of best practice in ESRD care and determine the future need for reaffirmation or reappraisal of the NICE RRT guidance.

## Data Availability

Not applicable.

## Abbreviations

APD: Automated peritoneal dialysis
aPD: staff assisted peritoneal dialysis
AVF: Arteriovenous fistula
CAPD: Continuous ambulatory peritoneal dialysis
EPS: Encapsulating peritoneal sclerosis
ESKD: End-stage kidney disease
HD: Home haemodialysis
HDF: Haemodiafiltration
HHD: Home haemodialysis
LV: Left ventricular
NICE: National Institute for Health and Care Excellence
PD: Peritoneal dialysis
PROMs: Patient reported outcome measures
RCT: Randomized control clinical trial
RRT: Renal replacement therapy
